# Development of Unity Simulator for Epidural Insertion Training for Replacing Current Lumbar Puncture Simulators

**DOI:** 10.7759/cureus.13409

**Published:** 2021-02-18

**Authors:** Joss Moo-Young, Timothy M Weber, Bill Kapralos, Alvaro Quevedo, Fahad Alam

**Affiliations:** 1 Faculty of Science, Ontario Tech University, Oshawa, CAN; 2 Faculty of Health Sciences, Ontario Tech University, Oshawa, CAN; 3 Software and Informatics Research Centre, Ontario Tech University, Oshawa, CAN; 4 Faculty of Business and Information Technology, Ontario Tech University, Oshawa, CAN; 5 Anesthesiology, University of Toronto, Toronto, CAN

**Keywords:** virtual simulation, epidural training, haptic feedback, makerspace

## Abstract

We have recently developed the Unity Simulator for Epidural Insertion Training (USEIT) system that provides an innovative and relatively inexpensive virtual simulation approach for epidural training. This report describes the design and development process to produce the USEIT system.

## Introduction

Epidural anesthesia is a regional anesthetic that blocks pain in a large area of the body and is performed by guiding a special needle into the epidural space in order to insert a catheter for the continuous infusion of local anesthetic [1]. It is the most popular method of pain relief during labor and used in 75% of childbirths in the United States [2]. The procedure itself is not without risk, and when performed incorrectly, it can result in insufficient pain relief, full-body anesthesia, severe headaches, back pain, bleeding, and nerve damage, and although more rare, other life-threatening complications can also occur [1,3]. A study that examined epidural procedures performed in the United Kingdom found that permanent harm from the procedure occurred in 3.1 to 6.1 in every 100,000 [4]. Performing the epidural procedure is difficult; one study showed that reaching epidural insertion competency requires between one and 85 attempts with many requiring 75 attempts [5], while another study found that only after 90 attempts was a success rate of 80% achieved [6]. As Isaacs et al. described, “such numbers may be difficult to achieve within defined training periods,” particularly given issues associated with teaching skill acquisition and the moving away from the apprenticeship model where trainees practice on patients [4,7]. The apprenticeship approach in anesthesia training and medical training, in general, is becoming less acceptable given the increasing focus on patient safety, along with budgetary constraints associated with teaching in a clinical environment, particularly when considering invasive procedures that require high-risk care [8]. Given these concerns, medical education has shifted to a simulation-based model that allows clinical situations to be replicated with various degrees of fidelity or realism, multimodality, immersion, and presence [9]. Simulation involves immersing the trainee in a realistic situation (scenario) created within a physical or virtual space (simulator) that replicates the real environment [10]. In the context of medical education, simulation can be defined as an education technique that allows interactive and immersive activity by recreating all or part of a clinical experience without exposing patients to the associated risks [11]. Simulation ranges from de-contextualized bench models in virtual reality (VR)-based environments that employ immersive technologies such as VR headsets, to high fidelity recreations of actual operating rooms [12]. Brazil et al. developed a virtual-reality-based simulator for epidural anesthesia training with the goal of reducing the high failure rate currently associated with the epidural procedure [13]. Their simulator includes three new models that provided greater realism than the prior simulator of Brazil et al.: (i) a force-displacement model to cover the progression of resistance forces generated by tissues along with the needle insertion, based on real experiment data; (ii) a force-needle model, to calculate the axial forces produced by needle deflection upon insertion, based on experiment data; and (iii) a tissue model to design the thickness for each tissue on the simulator, from patient height, weight and age, based on average body measurements [13]. The simulator environment implements the integration of a Phantom Omni haptic device to control the needle movement by the user. Gamification in the form of points and achievements (leaderboard) was also incorporated to improve user engagement. Future work will examine the effectiveness of their simulator. With respect to the epidural procedure, simulators have been available since the 1980s and have ranged from manikin-based simulators to computer-based simulators, to more recent simulators that incorporate immersive technologies such as virtual reality [14]. An overview of epidural simulators is beyond the scope of this technical report. However, a complete and thorough review that discusses 31 simulators, including details related to their hardware/software, and strengths and weaknesses is provided by Vaughan et al. [14]. It should be pointed out that there are advantages and shortcomings to each type of simulator, although, it has been suggested that serve its purpose, an epidural simulator must be of high technical fidelity at an affordable price [4].

This technical report describes the design and development of a virtual simulation, known as the Unity Simulator for Epidural Insertion Training (USEIT), for epidural training. The USEIT was developed leveraging visual and haptic feedback employing consumer-level (low-cost) technologies including the Novint Falcon haptic controller (Novint Technologies, Inc., Albuquerque, NM), and 3D printing in conjunction with open electronics. With respect to prior work, the USEIT simulation system is similar to the virtual reality simulator developed by Brazil et al. [13]. More specifically, both employ virtual reality (e.g., a virtual patient), and a haptic device to simulate the insertion of the epidural. However, the USEIT simulator employs a consumer-level haptic device (Novint Falcon) which is far more affordable than the Phantom Omni haptic device employed in the simulator of Brazil et al. [13]. Of course, affordability, fidelity, and accuracy are typically sacrificed; once validation data for both simulators are available, a comparison may reveal further insight regarding the benefits/limitations of low-fidelity simulators. 

## Technical report

USEIT development

The development process encompassed software and hardware components aimed at reproducing the epidural process. To determine the characteristics of USEIT, first, we defined the system’s inputs and outputs to perform the procedure. Inputs include: (i) capturing the position and orientation of the needle between the upper and lower lumbar spinous process, (ii) pushing the plunger flange to apply the solution in the syringe until loss of resistance (LOR), signifying entering the epidural space, is achieved, and (iii) menu inputs through the graphical user interface (GUI). Outputs include: (i) haptic feedback provided when inserting the needle and reaching the LOR, (ii) visual feedback providing information about the needle position and orientation towards the LOR, and (iii) user metrics for performance assessment after the simulation.

Software Design

**Figure 1:**
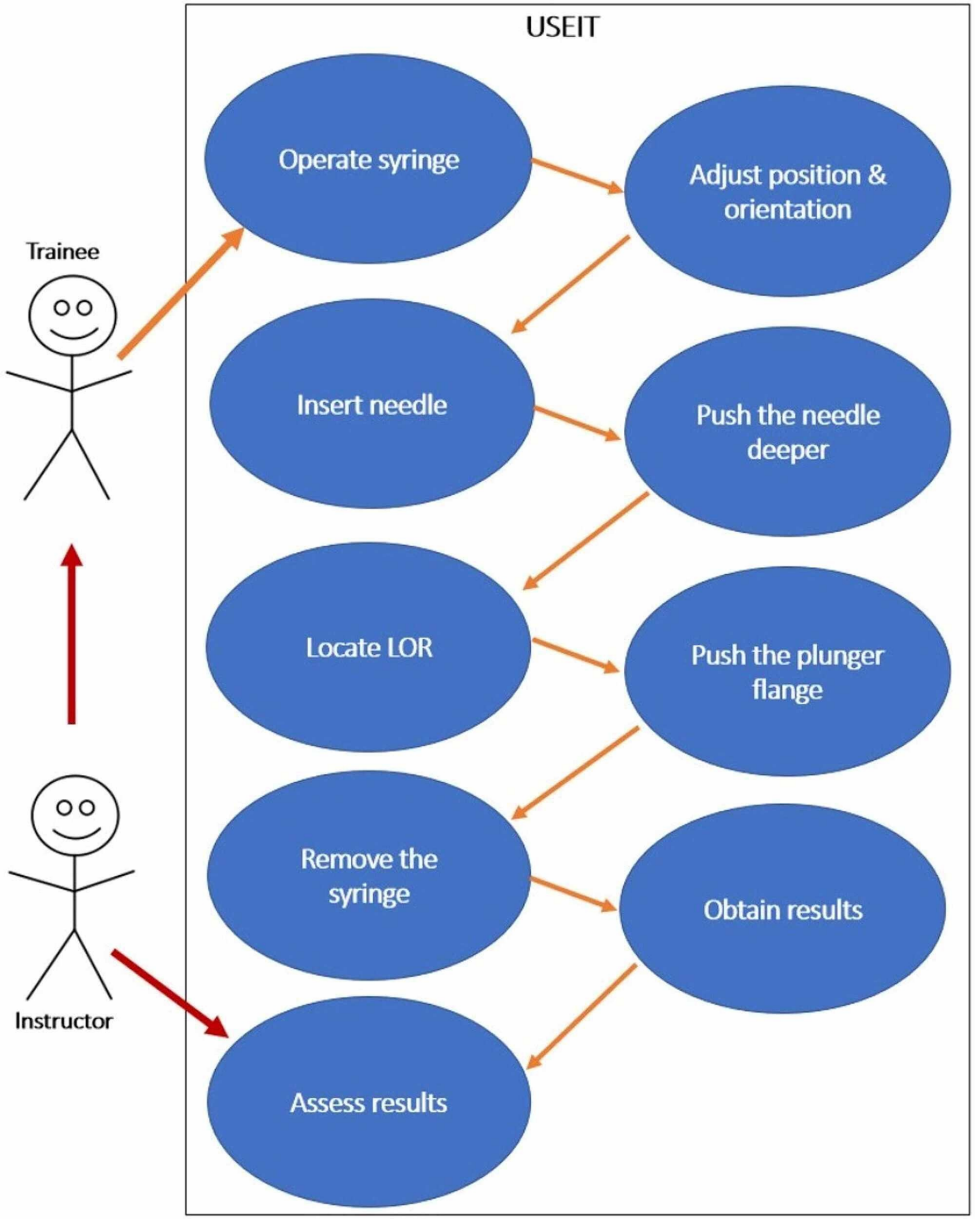
USEIT use cases. USEIT: Unity Simulator for Epidural Insertion Training

The software development process included the definition of uses cases for the trainee to perform as presented in Figure 1. The Unity game engine was chosen to develop the simulator for its cross-platform support and rapid prototyping features including asset integration, human interface devices compatibility, networking, and scripting.

Virtual Environment

**Figure 2:**
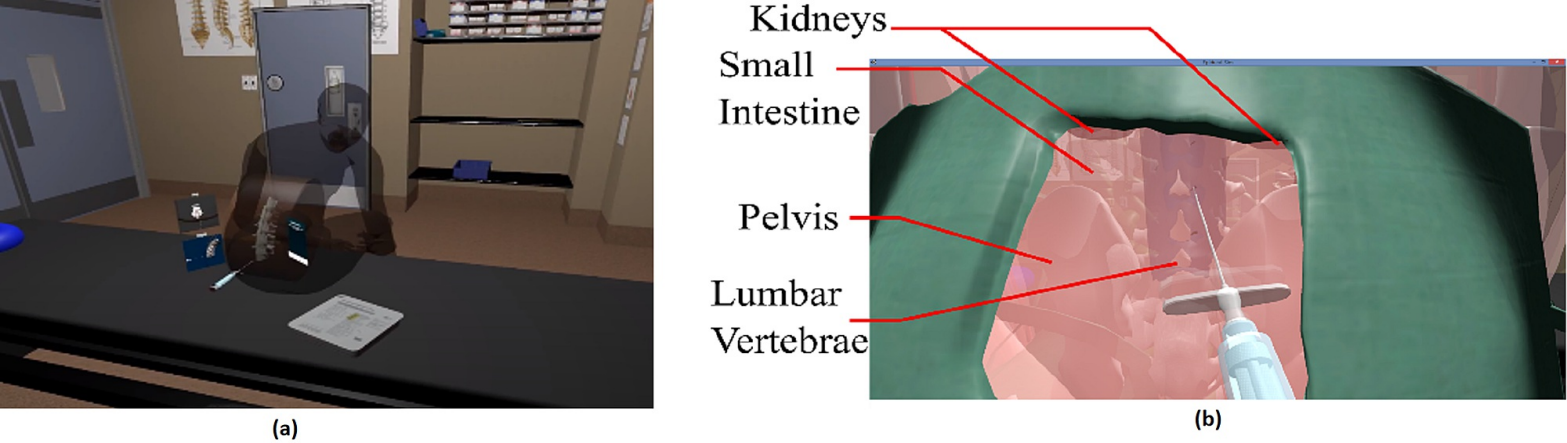
Virtual patient’s back displaying solid and transparent models for inserting the needle. (a) Patient in the "C" position. (b) Transparent view for locating the LOR. LOR: loss of resistance

The virtual environment was developed to resemble an operating room prepared for the epidural procedure. Additionally, a virtual patient in a C-shaped position ready for the procedure is shown in Figure 2a. For anatomical correctness, we employed computer models designed for medical training. Figure 2b shows a transparent view of the spinal column providing a view of the internal organs that must be accounted for when performing an epidural procedure.

Haptics Simulation

The Novint Falcon haptic gaming controller due to its affordability and technical properties summarized in Table 1. It should be noted that the device has been discontinued and there currently is no other comparable device available at the consumer level. The Novint Falcon was integrated into Unity using the C++ plugin employing the Chai3D haptic library, commonly used due to its accurate haptic algorithms [15]. Chai3D simulates 1000 frames per second (FPS), a value higher than that of the 60 FPS of Unity’s, thus ensuring that both visual and haptic feedback do not present delays that can affect the system response.

**Table 1:** Novint Falcon features.

Feature	Specification
3D Touch Workspace	4" x 4" x 4"
Position Resolution	> 400 dpi
Force Capabilities	> 2 lbs
Size	9" x 9" x 9"
Communication Interface	USB 2.0
Weight	6 lbs
Power	30 watts
Interchangeable Gripper	N/A

Gripper Customization

**Figure 3:**
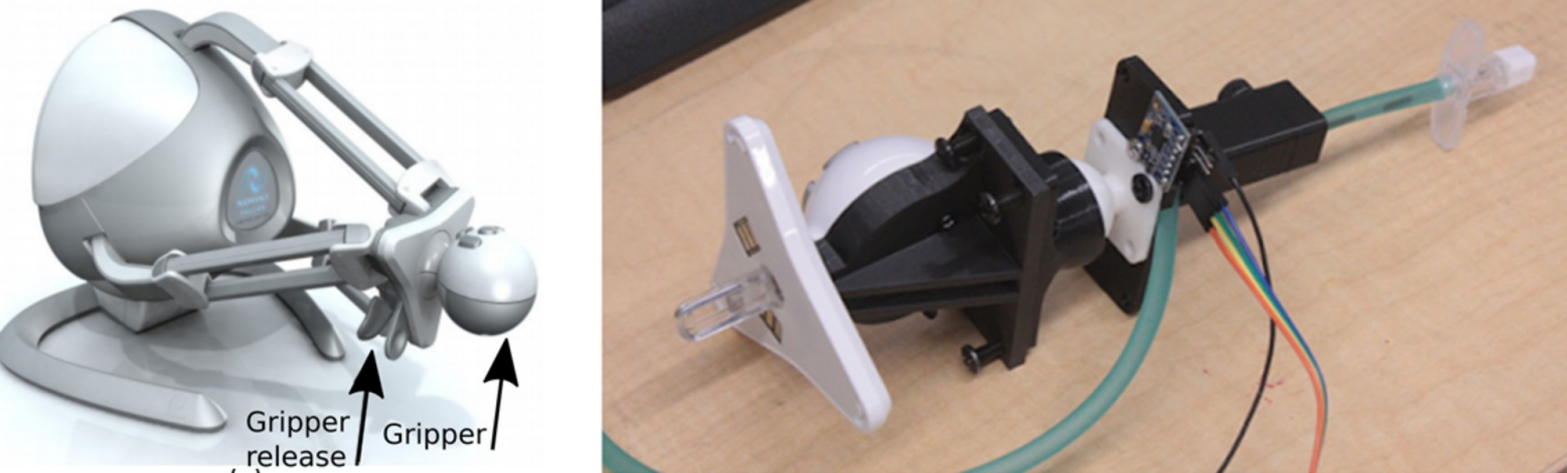
(a) Novint Falcon haptic device. (b) Custom-made (3D-printed) interface to connect the epidural needle to the haptic device.

The Novint Falcon allows its end-effector (“gripper”) to be changed (Figure 3a). Interchangeable grippers allow for user input customization ensuring that a wide variety of interactions. The epidural simulator requires a human interface device that resembles a real needle (i.e., high fidelity), ensuring that skills developed during training are transferrable to the real world [16]. We have designed a custom gripper replacement with the generic 4 mm screw Faceplate Adapter. The lower half of the Novint Falcon’s end-effector was removed and replaced with this adapter, and more specifically, the three degrees of freedom (3-DOF) ball joint is attached to the adapter using 4 mm screws (Figure 3b). Then the Touhy needle clamp is attached to the Ball Joint with 4 mm screws. Finally, a Touhy needle is connected to the tubing, and inserted into the front of the clamp. Two, hand-turned 4 mm bolts at the top of the clamp press down on the Touhy needle and tube to secure it firmly.

Orientation

A 6 DOF MPU6050 Inertial Measurement Unit (IMU) is attached to the custom-made gripper and controlled by a separate Arduino. Figure 3b shows the IMU connected with a ribbon cable. Data are sent from the Arduino to Unity by querying the yaw, pitch, and roll orientation of the needle, thus increasing the Novint Falcon’s DOF from three to six. 

Needle Force-Feedback Model

**Figure 4:**
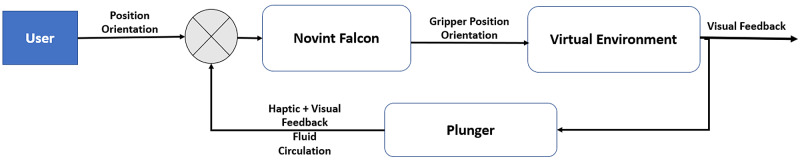
USEIT closed-loop system. USEIT: Unity Simulator for Epidural Insertion Training

The force feedback model was separated into the following two components to better simulate haptic interactions while inserting the needle to reach the LOR: (i) parallel to the needle direction and (ii) perpendicular to the needle direction. Each component is calculated separately and then combined into a 3D output force command for the haptic device. The haptic feedback is then used to alter the virtual environment by additionally providing visual feedback allowing the trainee to guide the needle towards the LOR. A closed-loop system, receiving position and velocity data from encoders in the haptic device, and orientation data from an additional orientation sensor, is employed to create the interactive loop for the trainee to practice the procedure while getting feedback as presented in Figure 4.

Parallel Force Component

**Figure 5:**
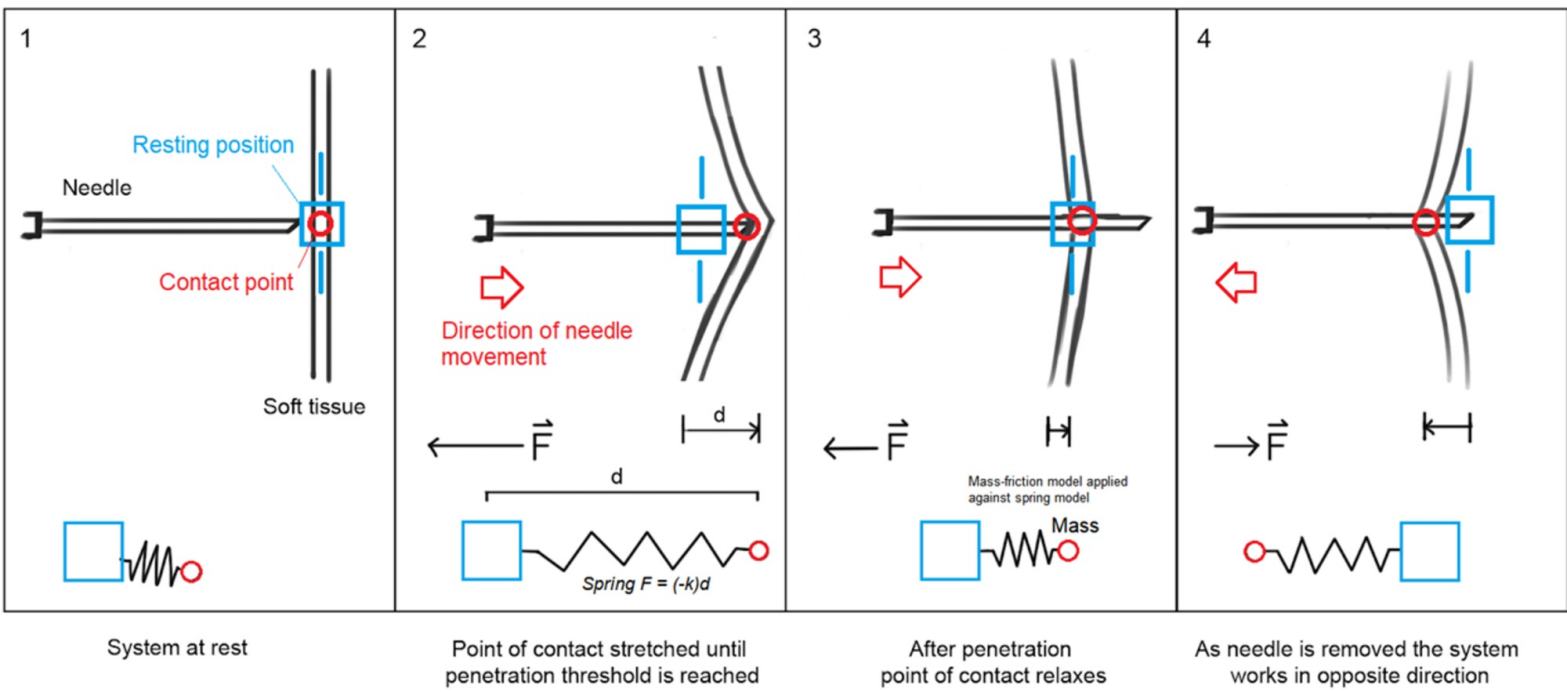
Mass-spring model for needle insertion simulation.

The geometry of each haptic-enabled object in the simulation is sent from Unity to Chai3D as either solid (e.g., bone) or penetrable (e.g., skin or ligament). During the procedure, a ray cast (i.e., a virtually projected line) determines the type of haptic collider object in the needle’s path. This translates into a one-dimensional array of tissue layers sorted by depth. In this model, two points are tracked: i) the contact point between the needle and the layer, and ii) the resting position of the layer under zero stress. For each layer, tension and friction effects are simulated employing a mass-spring-friction model to provide the sensation of needle insertion. Figure 5 shows a visual representation of the mass-spring model during the different stages of penetrating the layers.

Each tissue layer includes fluid resistance, penetration force, and friction properties to increase realism. The spring stiffness value (k) was configured to mimic the force required to penetrate, the contact point between the needle and the layer [17]. The spring’s force applied to the contact point and at the resting position allows calculating the output force to be generated by the USEIT system for haptic feedback when the needle is pressed against a layer. After the force threshold is reached, the contact point is simulated as a mass, capable of moving back towards the resting position of the layer, pulled by the spring.

Perpendicular Force Component

The perpendicular force model is applied when the needle enters a penetrable object such as skin. Since the needle orientation takes place within the 3D virtual space, we employed a 2D plane perpendicular to the needle direction to output a controlled force focused on the needle entry point. The position of the needle during the procedure in conjunction with that of the entry point are both projected onto the 2D plane to control the amount of force generated. The force response is controlled by a Proportional Integral Derivative (PID) controller, which takes this position as input and outputs a controlled force in 2D toward the needle entry point. A PID controller is a control loop mechanism employing feedback, which is used to compare and modulate outputs [18].

Plunger and Fluid Resistance

**Figure 6:**
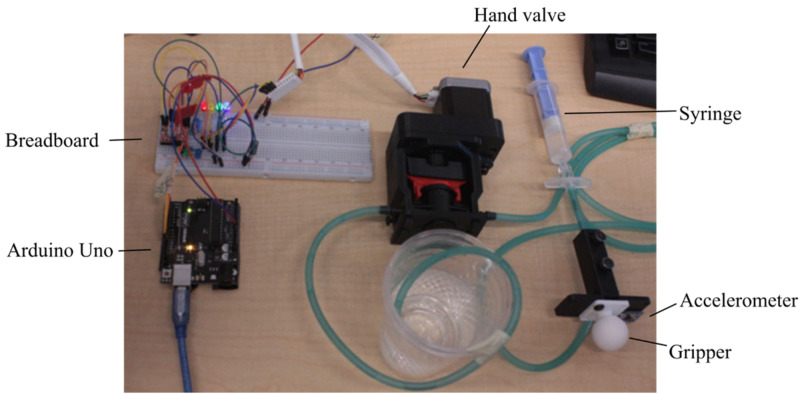
Custom-made gripper and hand valve.

 Part of the epidural procedure is the anesthetic administration, and to simulate this, a hand valve is fitted into a stepper motor that is connected to the syringe coupling. More specifically, a NEMA 17 size stepper motor is connected through a 1:4 gearbox with 2.5 mm bolts. The gearbox exists solely to make the rotation of the valve smoother and more reliable. Next, the stepper motor is connected (via a 6-pin connector) to an A9488 stepper motor driver with a 12 V power supply. The motor driver’s current limiting potentiometer is trimmed according to the specification of the motor. Finally, the stepper motor’s step-per-revolution is configured in the Arduino driver code. A 200 step-per-revolution motor is treated as 800 step-per-revolution due to the gear ratio. Using a hand valve also allows for partial opening and closing for varied levels of fluid resistance. In Unity, the fluid resistance control software queries the haptic simulation for data on the layer and the tip of the needle within, then uses its fluid resistance property to adjust the resistance level, normalized between zero (closed) and one (fully open). An overview of this system is provided in Figure 6. Note that the plastic cups are employed to allow for the fluid to be recirculated.

## Discussion

Here, we discuss the areas of improvement for USEIT. Although formal testing has yet to conduct, we have presented USEIT to many content experts and interested stakeholders at numerous medical-based conferences, workshops, and meetings. including Simulation Canada’s Sim Expo 2019 (October 21-22, 2019 in Montreal, Canada) and 2019 IEEE International Conference on Consumer Electronics (ICCE) [19]. This informal feedback has allowed us to identify shortcomings and areas requiring further improvement.

Fidelity

Here, the attention focused on two main areas, technology limitations, and the current implementation. With respect to technology limitations, occasionally the USEIT system physically oscillates the needle, distracting the user and breaking immersion. This occurs when the force applied to the system exceeds a constraint within the virtual environment, or when the device loses calibration. If immersion is broken, the realistic perception lowers as the system behaves in an unrealistic manner. This problem lies within the Novint Falcon libraries and how they handle large forces. Unfortunately, we have not found a solution for this as the same situation occurs with other haptic plugins. Calibration is performed every time the system is used to reduce this jittering. Regarding the current implementation, the use of the custom-made gripper and the hand valve was found innovative, reproducible, and meaningful with respect to increasing realism within the procedure. However, palpation of the ‘patient’s back’, which is a key step to determine the point of insertion was found missing during our demo sessions. Palpations were omitted from our simulation since adding another custom-made gripper for adequate palpation would have increased the system complexity as trainees would have to engage in assembling and disassembling the system, which can lead to malfunctions from wear and tear.

Design

**Figure 7:**
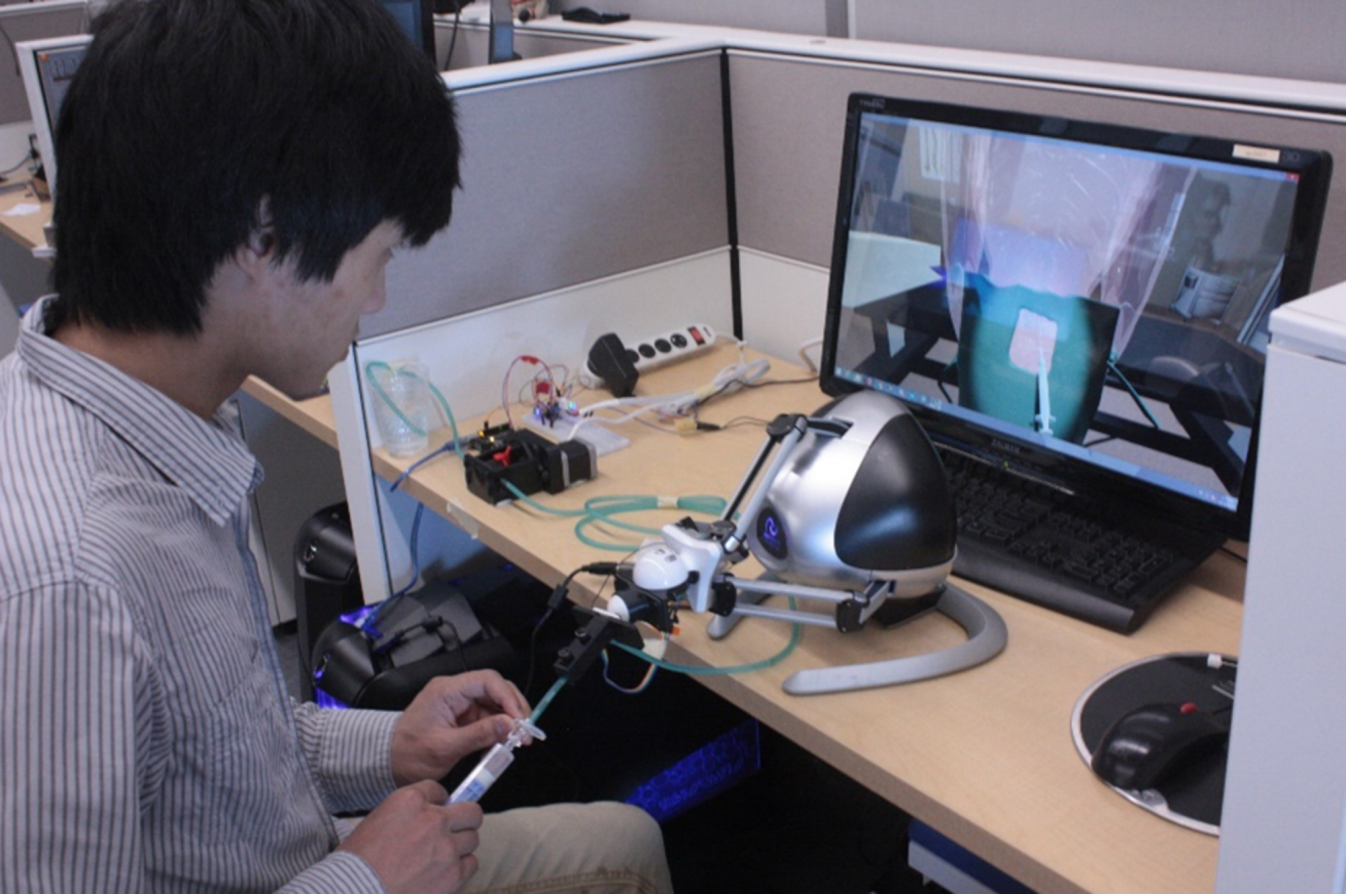
User performing the epidural procedure with USEIT.

Here, the attention focused on the hardware and virtual environment design. On the hardware side, the USEIT system is a mosaic of different components connected together. At this point, all interactive components have been designed to mimic the epidural process. However, we are currently investigating alternative components, while troubleshooting the existing model to improve the 3D printing process to secure all electronic components and ease assembly and use. Figure 7 presents a user employing the system, tilting the needle to insert it. On the software side, the virtual environment was found adequate and immersive. However, in its current state, it is missing improvements geared towards training assessment other than orientation and time completion metrics.

 Ergonomics

**Figure 8:**
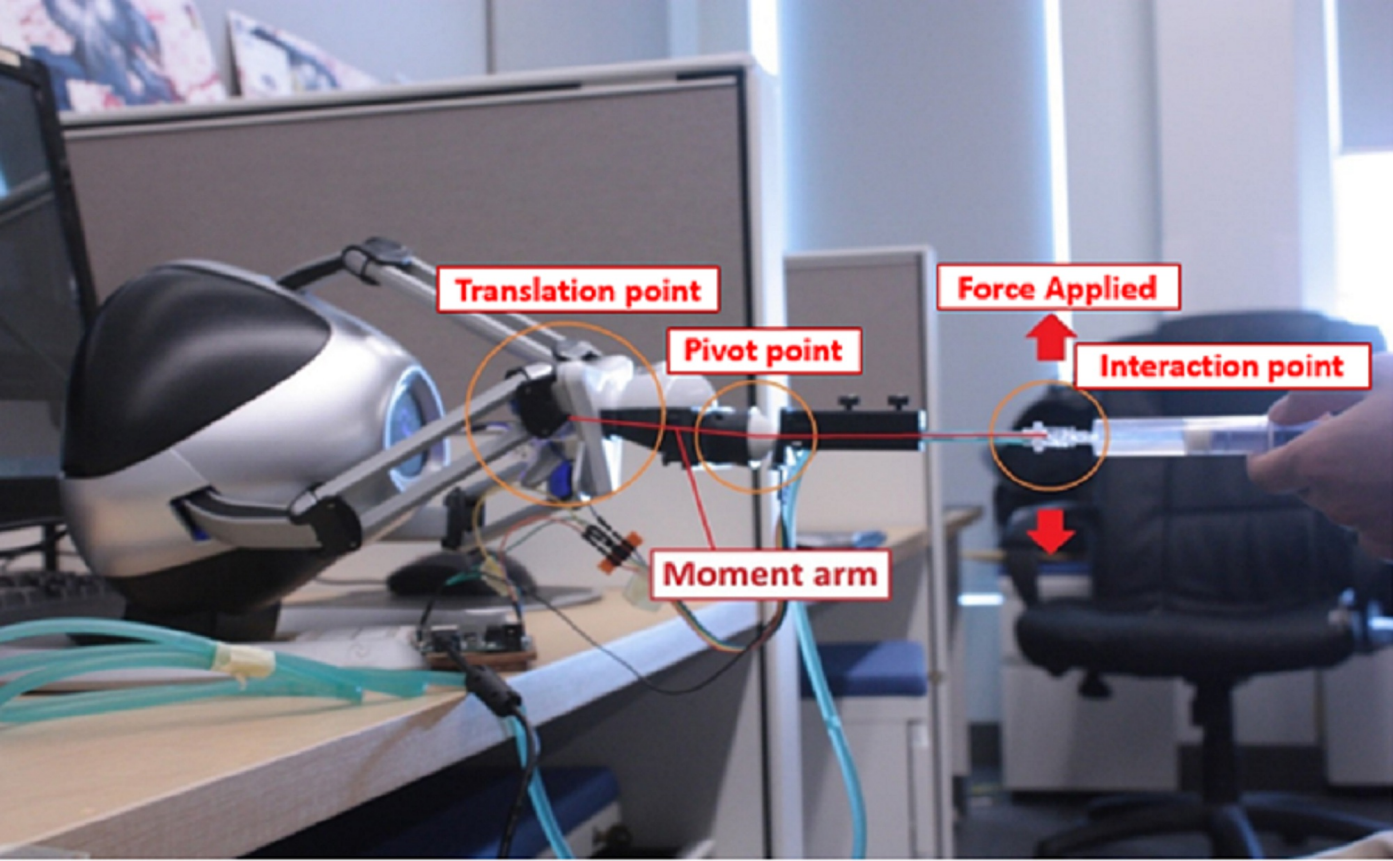
Distance between the syringe and the interaction point.

Due to the quantity of space required by the gripper, USEIT distances the user from the screen, thus creating a gap that is unrealistic when compared to the real procedure, where the back of the patient is closer to the syringe (Figure 8). The distance can increase the difficulty to operate the syringe with smoothness. We are working on a more optimized hardware design that may minimize this moment arm to reduce or eliminate the issue. Moreover, the use of a screen reduces immersion due to the gap between the trainee and the virtual environment. To this end, we are currently exploring the addition of virtual and mixed reality to bring the content closer to the user, similarly to real life.

## Conclusions

Here, we have presented USEIT, a virtual epidural training system that employs affordable consumer-level technologies, and we have focused on the development process employing consumer-level technologies. Approaching USEIT prototyping from an accessibility and affordability point of view, allowed us to implement and explore shortcomings and opportunities of a haptics game controller, 3D printing, and open electronics. While all the components of USEIT are important, the Novint Falcon is the foundation of the project as at the time of developing the system it provided us with a set of technical features adequate for simulating the epidural process. While the Novint Falcon provides a suitable haptic user interface, we had to customize the gripper to add three additional degrees of freedom to allow us to track the orientation of the needle, in conjunction with the hand valve to enable pushing fluid through the syringe once attached to the gripper. On the software side, CHAI3D provided a reliable simulation model for replicating the needle insertion, which we accomplished by setting a set of layers and combined forces towards achieving LOR when reaching the epidural space. Furthermore, Unity provides a suitable game engine to create an immersive environment that represents appropriate visuals for recreating the procedure digitally. Finally, this process has shown that it is possible to develop such consumer-level systems in a manner that presents potential solutions to lower simulation costs that can help practice medical skills.

Future directions

The next phase of development will include face, content, and construct validity testing. In addition, expert opinions gathered during our informal testing sessions will be integrated to create an iteration of USEIT that will include both palpation and landmarking. The representation of what is happening in real-time will still be included on a computer monitor, for pedagogical purposes. Furthermore, we will explore virtual and mixed reality to increase immersion in addition to gamification to increase engagement and motivation.
